# An adaptive transformer-based framework for advanced brain activity mapping and intelligent neurotherapeutic decision support

**DOI:** 10.3389/fnhum.2025.1551168

**Published:** 2025-10-02

**Authors:** Bhushankumar Nemade, Vikram Kulkarni, Deven Shah, Keyur Patel, Shreyaskumar Patel, Uma B. Goradiya

**Affiliations:** ^1^Shree L. R. Tiwari College of Engineering, Mumbai University, Mumbai, India; ^2^Department of Information Technology, Mukesh Patel School of Technology Management and Engineering, SVKM’s Narsee Monjee Institute of Management Studies, Mumbai, India; ^3^Terna Engineering College, Faculty of Science and Technology, University of Mumbai, Mumbai, India; ^4^New Jersey Public Health Association, New Brunswick, NJ, United States; ^5^NetScout Systems, Inc., Allen, TX, United States

**Keywords:** EEG signal analysis, transformer architecture, neurotherapeutic decision support, temporal-spatial modeling, precision brain imaging, adaptive attention mechanism, machine learning in neurology

## Abstract

**Introduction:**

Identification and treatment of neurological disorders depend much on brain imaging and neurotherapeutic decision support. Although they are loud, do not remain in one spot, and are rather complex, electroencephalogram (EEG) signals are the principal tool used in research of brain function. This work employs an Adaptive Transformer-based technique with improved attention processes to extract temporal and spatial relationships in EEG data, effectively addressing these issues.

**Methods:**

First processed to eliminate noise and split them into time-series chunks, EEG data are then included into the proposed approach. Channel-wise embeddings and temporal encoding help to depict the data. Then, a transformer design including spatial attention for inter-channel interactions, multi-head self-attention for temporal aspects, and an adaptive attention mask for domain-specific modifications is used. Other openly accessible EEG datasets as well as the TUH EEG Corpus and CHB-MIT were evaluated against the model. Its performance was scored using metrics like accuracy, precision, memory, and F1-score.

**Results:**

The suggested method was more accurate than standard models like CNNs and LSTMs, with a score of 98.24%. The method was also shown to be able to find minor patterns in EEG data by improving precision and memory. Attention maps showed important areas of time and space, which made them easier to understand and useful in professional settings.

**Discussion:**

The Adaptive Transformer turns out to be a useful tool for neurotherapeutic use of EEG data modeling. The approach provides greater medical assistance and knowledge on the functioning of the brain as well as answers significant issues. Future research might focus on subject-specific modifications and interaction with real-time systems.

**Conclusion:**

This study demonstrates the potential of transformer-based models in revolutionizing EEG analysis for precision brain imaging and neurotherapeutic decision-making.

## 1 Introduction

Understanding brain function and detecting neurological diseases has long depend on electroencephalography (EEG), a fundamental instrument. EEG offers real-time understanding of brain dynamics by gathering electrical activity using non-invasive electrodes positioned on the head. Its uses are many and range from monitoring brain-computer interface systems to diagnosis of epilepsy and cognitive state evaluation. Because of its great temporal resolution, which makes it essential for research on the complex temporal and spatial patterns of the brain, EEG is crucial in precision brain imaging. Nevertheless, given the natural complexity of the signals, EEG analysis presents significant difficulties even with its promise (Ajali-Hernández et al., 2024; Dong et al., 2024).

Usually impacted by both internal and external events, EEG signals are famously non-stationary–that is, their statistical characteristics shift with time. This feature makes the identification of significant trends difficult as conventional approaches find difficulty to adjust to such dynamic changes. Another major obstacle is noise as ambient interference, muscle movements, or artifacts from eye blinks can contaminate EEG recordings. Furthermore very complicated temporal and spatial connections seen in EEG data need for sophisticated analysis methods to reveal underlying patterns. These difficulties have spurred a lot of study on more advanced computer approaches for EEG analysis (El Hadiri et al., 2024; Gunia et al., 2024).

Although conventional machine learning and deep learning models have been extensively used in EEG signal processing, they have natural restrictions. Support vector machines or random forests are two examples of classical machine learning methods that mostly depend on feature engineering, which calls for domain knowledge and usually misses the whole complexity of EEG signals. By automating feature extraction and developing hierarchical representations, deep learning models–CNN and LSTM among others–have shown a significant improvement (Ibitoye et al., 2021; Khayretdinova et al., 2024). These models, too, have limitations. CNNs are great in extracting spatial characteristics, but they often ignore the temporal dynamics that are very important in EEG analysis. On the other hand, LSTMs struggle with spatial connections between EEG channels while specializing in modeling sequential data. Usually requiring huge datasets to properly generalize, both methods are prone to overfitting when used on smaller, domain-specific datasets. Moreover, their interpretability is still restricted, which makes clinical adoption difficult when explainability is essential (Kim et al., 2024; Klooster et al., 2024).

With these constraints, the development of transformer-based models offers a transforming answer. Originally designed for “natural language processing,” (NLP), transformers with their attention techniques have transformed sequential data processing. Transformers are especially fit for complicated temporal and spatial modeling as they can record long-range relationships within data unlike conventional recurrent or convolutional architectures. Transformers’ fundamental invention is their self-attention mechanism, which gives input components dynamic weights according on their significance for the current job. This adaptability lets transformers concentrate on the most useful sections of the data, which fits very well with the difficulties of EEG signal analysis (Li et al., 2024). Transformers-based models have shown amazing success in different fields. Models like BERT and GPT have established new standards in NLP by deftly grasping semantics and context. Using both global and local information, transformers have outperformed conventional CNNs in computer vision applications like object identification and picture categorization. Time-series analysis, in which transformers have been used to anticipate financial patterns, track industrial processes, and project medical results, has likewise evolved from their usage. These developments show the adaptability and possibilities of transformers, therefore motivating their use in EEG analysis (Mutawa and Hassouneh, 2024).

Transformers’ capacity to solve important problems drives their acceptance for EEG study. Overcoming CNN and LSTM, their self-attention method can concurrently represent spatial linkages and temporal dynamics. Furthermore, transformers are naturally scalable and can handle big and complicated datasets free from sequential computation’s limitations (Dong et al., 2024; El Hadiri et al., 2024). For EEG research, where the data volume might be significant and real-time processing is usually needed, this scalability is very helpful. Moreover, transformers provide better interpretability than conventional deep learning models. Visualizing attention weights helps researchers and doctors understand which areas of the EEG data best support the predictions of the model, therefore strengthening confidence and enabling clinical decision-making.

This work aims to provide a unique adaptive transformer-based method for neurotherapeutic decision assistance and precision brain imaging. The following are our novel contributions:

- Using a domain-specific adaptive attention mask, the adaptive attention mechanism dynamically focuses on important temporal and spatial EEG characteristics for EEG variance. By stressing important areas and reducing noisy or pointless data, this approach guarantees consistency and resilience across subjects and situations. This approach targets significant patterns and reduces brainwave data variance, therefore enhancing the accuracy of EEG analysis.
- Integrating transformers for temporal-spatial modeling: Transformers endowed with “multi-head self-attention” record long-range temporal interdependence in EEG channels. Analyzing inter-channel interactions with spatial attention offers a whole temporal-spatial awareness of EEG data. This integration models concurrently time-based dynamics and spatial interdependencies, therefore enabling the whole interpretation and analysis of an EEG signal.
- Positional encoding provides an end-to-end framework to retain sequential structure of the EEG signal by means of feature embedding framework. Transformers convert raw EEG data into high-dimensional channel-wise embeddings, therefore effectively processing it. Through analysis of intricate temporal-spatial patterns in EEG data, this paradigm clarifies and guides application of brain activity dynamics.

This work offers many different kinds of contributions. It first presents a new framework using transformer architecture to replicate the complex spatial and temporal dynamics of EEG signals. Second, it visualizes attention weights to close the interpretability gap in EEG data and offers doctors practical insights. Third, it shows how scalable and flexible transformers are in managing various EEG datasets, hence opening the path for individualized neurotherapeutic uses. This work attempts to improve the accuracy, efficiency, and usefulness of EEG-based diagnoses and treatment by bridging the gap between modern computational approaches and clinical demands.

In EEG analysis for precision brain imaging and neurotherapeutic decision assistance, the suggested adaptive transformer-based method marks a major advance. This study introduces an adaptive transformer-based model that enhances EEG signal analysis by capturing complex spatial and temporal relationships. The proposed approach improves neurotherapeutic decision support and enables better precision in brain imaging. By integrating domain-specific modifications, it ensures robust and scalable performance across diverse EEG datasets. The combination of innovative models which include transformers with domain-specific knowledge offers excellent capability for commencing new horizons in neuroscience and clinical practice as the field develops.

## 2 Literature review

The study of brain dynamics through EEG analysis has become increasingly significant, offering a window into the functioning and disorders of the human brain. EEG provides a non-invasive, cost-effective approach to understanding brain activity, facilitating advancements in neurological diagnosis and therapeutic interventions. Despite its potential, the complexity of EEG signals, influenced by non-stationary characteristics and noise, presents substantial challenges for effective analysis. Advanced computational techniques, particularly those incorporating machine learning and deep learning, have been extensively employed to address these challenges, yet limitations remain.

Efforts to utilize self-supervised learning in EEG analysis have demonstrated promising outcomes, particularly in brain disease diagnosis, where adaptive node feature extraction has been shown to improve classification accuracy. This approach highlights the need for advanced modeling techniques capable of leveraging the hierarchical nature of brain networks (Zeng et al., 2024). Similarly, genetic variations influencing oscillatory alpha power in EEG data have been linked to specific neurobiological mechanisms, suggesting the potential for personalized models in precision brain imaging (Tichelman et al., 2023). The introduction of growth charts for brain function from infancy to adolescence provides a benchmark for understanding neural development, offering a reference point for the detection of anomalies (Iyer et al., 2024).

Deep learning-based frameworks have emerged as a dominant paradigm for EEG analysis, leveraging their ability to capture intricate temporal and spatial patterns. For example, research centered on classifying ADHD through deep learning methods have highlighted the role of particular mind regions inside the sickness, demonstrating the capability of neural networks in localizing pathophysiological markers (Sanchis et al., 2024). Also, reusable benchmarks for mind-age prediction have showcased the software of resting-state EEG signals in identifying age-associated neural changes, presenting a robust platform for exploring brain health throughout the lifespan (Engemann et al., 2022).

The hierarchical organization of complex correlation patterns in the brain has been effectively modeled to define a functional architecture, advancing the understanding of brain dynamics. Such models contribute to the identification of robust biomarkers for neurological conditions (Varga et al., 2024). Comparisons between EEG and MRI for early detection of “cortical dysmaturation” have highlighted the complementary nature of these modalities, emphasizing the need for integrative approaches in neuroimaging (White et al., 2024). The engineering of pluripotent stem cells for monitoring brain function and controlling neural activities further illustrates the interdisciplinary potential of EEG analysis (Cheng et al., 2024).

Sleep studies have also benefited from EEG biomarkers, particularly in understanding the activation of the brain’s lymphatic drainage system and its association with the blood-brain barrier. This opens avenues for exploring the therapeutic implications of sleep on neurological health (Semyachkina-Glushkovskaya et al., 2023). Meanwhile, transformer-based models have recently gained attention for their applicability in brain-computer interfaces, leveraging attention mechanisms to enhance the analysis of EEG signals (Pfeffer et al., 2024). Hybrid approaches combining EEG with other modalities like “functional near-infrared spectroscopy” (fNIRS) have further enriched the analytical capabilities, particularly in tasks requiring multidimensional feature extraction (Maher et al., 2023).

Scales Food and environmental elements have been shown to be sensitive by EEG studies on how outside events influence brain responses (Wu et al., 2022). Moreover, a novel approach linking visual stimuli to neural responses for cognitive analysis is stimulus-evoked EEG manifold learning for neural image classification (Falciglia et al., 2024). Finding indicators unique to the brain has demonstrated benefit from machine learning approaches. This makes more individualized therapies possible as well as more accurate diagnosis (Bomatter et al., 2024).

There are still issues even with these developments. Because EEG data is not always the same and has many dimensions, many times classic machine learning and deep learning models such CNNs and LSTM networks struggle with it. These techniques overfit, particularly in cases of noise presence, and need a lot of feature engineering to be done by hand. Furthermore, they are more difficult to grasp and use in different contexts as people are not always aware of the structural and geographical connections included within EEG patterns.

Originally designed for natural language processing applications, transformer-based models have developed a fresh approach of viewing EEG data. Long-range links and contextual linkages are quite well captured by these models. This qualifies them ideal for handling complicated in terms of time and spatial EEG data. Many fields have seen effective use of transformer designs as they can adapt to various data types and uncover interesting trends.

Building on these concepts, this work proposes to use a technique especially intended to operate with EEG data based on flexible transformers. This approach adds domain-specific modifications, such as a flexible attention mechanism, aiming to solve the issues with present models. This should make neurotherapeutic decision support and brain imaging more exact. This approach not only models complex data using the best characteristics of transformers but also provides us with a means to make EEG analysis more scalable and understandable. With this novel concept, the research aims to close the gaps in present approaches and therefore advance precision medicine and tailored brain care.

## 3 Methodology

**FIGURE 1:**
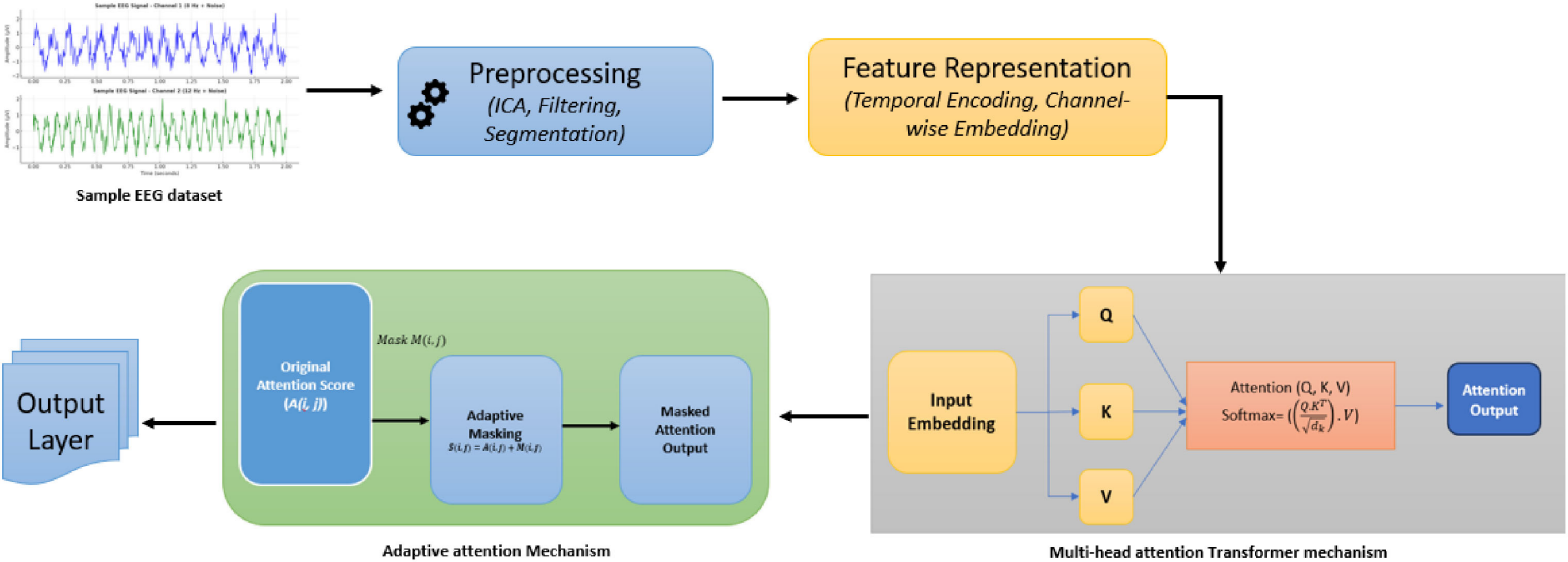
Proposed system architecture.

As shown in the following Figure 1, The TUH EEG Corpus dataset was used in this study, providing a diverse collection of labeled EEG recordings that facilitate model evaluation across various neurological conditions, including seizure detection and cognitive state analysis. In continuation it included in this study are noise and non-stationarity’s related problems. The five basic steps that constitute the approach that has been described are preprocessing of the data, feature representation, transformer design, integration of adaptive attention masks, and the output layer customized for neurotherapeutic predictions. Every stage uses the use of mathematical ideas-based sophisticated methods in signal processing and machine learning.

### 3.1 Experimental setup

To assess the impact of hyperparameter variations, an ablation study was conducted by adjusting key parameters:

- Number of attention heads: Models were evaluated with 4, 8, and 12 heads, showing optimal performance at 8 heads.
- Model depth: 3-layer and 6-layer configurations were tested, with 6 layers achieving the best balance of accuracy and computational efficiency.
- Learning rate: A range from 0.0001 to 0.005 was explored, with 0.001 yielding optimal stability and convergence.

### 3.2 Dataset

The “TUH EEG Corpus” dataset was used in this study, providing a diverse collection of labeled EEG recordings that facilitate model evaluation across various neurological conditions, including seizure detection and cognitive state analysis. This dataset comprises labeled EEG signals spanning a broad spectrum of neurological illnesses, including cognitive assessments and seizure detection. Preprocessing the data helps to remove noise and artifacts, thereby ensuring the high quality of the input for the training and model testing. Their great number of temporal and spatial patterns makes them ideal for assessing novel transformer-based algorithms in neurotherapeutic uses.

### 3.3 Data preprocessing

Electroencephalogram signals are inherently noisy due to artifacts from muscle movement, eye blinks, and external interference. Preprocessing ensures that only relevant neural information is retained. The EEG data used in this study was recorded using the NeuroScan SynAmps2 EEG system with a 64-channel cap following the 10–20 electrode placement system, ensuring standardized spatial coverage. EEG signals were sampled at 256 Hz, and preprocessing included:

- Artifact Removal: techniques such as Independent Component Analysis (ICA) are employed to separate noise from neural signals as shown in Equation 1.

(1) X=A.S

where *X* is the “observed EEG data”, *A* is the” mixing matrix”, and *S* is the “source signal matrix”. ICA estimates S by maximizing independence among components.

- Filtering: Band-pass filtering retains frequencies within a specific range (e.g., 0.5–50 Hz) used to remove low-frequency drifts and high-frequency noise as shown in Equation 2.

(2) Y⁢(f)=H(f).X⁢(f)

where *H(f)* is the “filter response” and *X(f)* is the “input signal” in the frequency domain.

- Segmentation: Signals are divided into “*fixed-length time windows*” (T) to capture temporal patterns, as shown in Equation 3, where *t_0_* is the “start time of the window”, Signals were divided into 2-second epochs to capture relevant temporal patterns

(3) $$S_{w}\,\left(t\right)=\left\{s\,\left(t\right)|t_{0}\leq t\leq t_{0}+T\right\}$$

### 3.4 Feature representation

**FIGURE 2:**
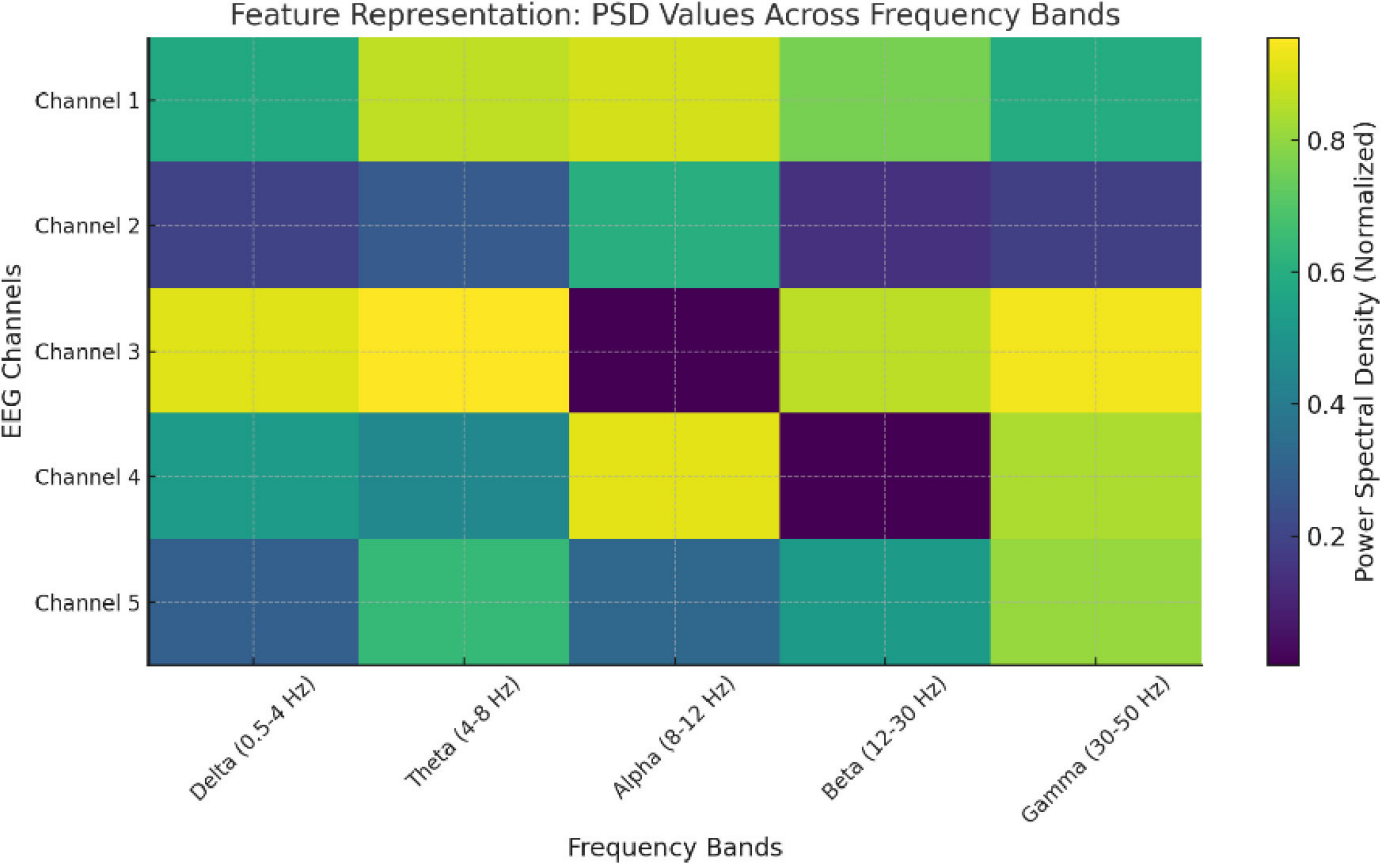
Feature representation: PSD values across frequency bands.

To make EEG signals compatible with transformer models, they are converted into structured, high-dimensional embeddings as shown in Figure 2 and Table 1.

**TABLE 1:** Feature representation of EEG data.

EEG channel	Delta (0.5–4 Hz)	Theta (4–8 Hz)	Alpha (8–12 Hz)	Beta (12–30 Hz)	Gamma (30–50 Hz)
Channel 1	0.5824	0.8669	0.8959	0.7605	0.5955
Channel 2	0.1993	0.2766	0.6028	0.1393	0.184
Channel 3	0.9114	0.9563	0.0052	0.8625	0.938
Channel 4	0.5257	0.4489	0.9188	0.0075	0.8405
Channel 5	0.2966	0.6412	0.3232	0.5229	0.8083

- Temporal Encoding: Position within the time-series sequence is encoded to retain temporal dependencies as shown in Equations 4, 5. A sinusoidal function is used

(4) $$P\,E_{p\,o\,s,2\,i}=s\,i\,n\,\;\left(\frac{p\,o\,s}{10000^{\frac{2\,i}{d}}}\right)$$

(5) $$P\,E_{\left(p\,o\,s,2\,i+1\right)}=c\,o\,s\,\;\left(\frac{p\,o\,s}{10000^{\frac{2\,i}{d}}}\right)$$

where pos is the “position”, i is the “dimension index” and d is the “embedding size”.

- Channel-wise embedding: Each EEG channel is treated as a separate sequence, and a linear embedding maps it to a higher-dimensional space, as shown in Equation 6.

(6) $$E_{c}=W_{c}.S_{c}+b_{c}$$

### 3.5 Transformer architecture

The core of the proposed method is the transformer, which effectively models temporal-spatial relationships in EEG data. The proposed model is configured as follows:

- Layers: 6 Transformer encoder layers, balancing depth and computational efficiency.
- Attention heads: 8 multi-head attention units, ensuring effective feature extraction.
- Embedding size: 256 dimensions, capturing rich temporal-spatial representations.
- Feedforward network: Two fully connected layers with ReLU activation for non-linear transformations.

Ablation studies confirmed that increasing depth beyond 6 layers led to diminishing gains, while 8 attention heads provided the best trade-off between complexity and performance.

- Multi-head self-attention: Captures dependencies across time within each channel where Q,K,V are “query, key and value” matrices derived from input embeddings as shown in the following Equation 7

(7) $$A\,t\,t\,e\,n\,t\,i\,o\,n\,\left(Q,K,V\right)=s\,o\,f\,t\,m\,a\,x\,\left(\frac{Q.K^{T}}{\sqrt{d_{k}}}\right).v$$

- Feedforward layers: Each attention output is processed through a position-wise feedforward network, as shown in Equation 8, where *W*_1_,*W*_2_ are weights and *b*_1_,*b*_2_ are the biases:

(8) $$FFN\left(x\right)=ReLU\left(W_{1}.x+b_{1}\right).W_{2}+b_{2}$$

- Spatial attention: Captures inter-channel relationships as shown in Equation 9:

(9) $$A_{s}\,\left(i,j\right)=\frac{\text{exp}\,\left(e_{i\,j}\right)}{\sum_{k=1}^{C}\text{exp}\,\left(e_{i\,k}\right)}$$

where *A*_*s*_ (*i*,*j*) represents the spatial attention weight between channel *i* and channel *j*, *e*_*ij*_ is the similarity score, and *C* is the total number of channels.

### 3.6 Adaptive attention mask

Electroencephalogram variability across subjects and conditions necessitates domain-specific adjustments. The adaptive attention mask emphasizes critical temporal-spatial regions dynamically.

- Dynamic masking: A learned mask *M* is applied to attention scores, as shown in Equation 10. Here *A*_(i,j)_ is the original attention score, and *M*_(i,j)_ is the adaptive mask weight.

(10) $$\hat{A}\,\left(i,j\right)=A\,\left(i,j\right).M\,\left(i,j\right)$$

- Optimization of mask: Masks are trained using a loss function that penalizes irrelevant regions as shown in Equation 11.

(11) $${ℒ}_{m\,a\,s\,k}=\underset{i,j}{\sum }\left(1-M\,\left(i,j\right)\right).A\,\left(i,j\right)$$

### 3.7 Output layer

The final layer converts the transformed embeddings into actionable predictions, either classification or regression outputs.

- Classification: A softmax function maps the output to probabilities for each class as shown in Equation 12,

(12) $$P\left(y=k|x\right)=\frac{\text{exp}\left(W_{k}.h+b_{k}\right)}{\sum_{j=1}^{K}\text{exp}\left(W_{j}.h+b_{j}\right)}$$

where *h* is the hidden representation, *W_k_* and *b_k_* are the weight and bias for class *k*, and *K* is the total number of classes.

**FIGURE 3:**
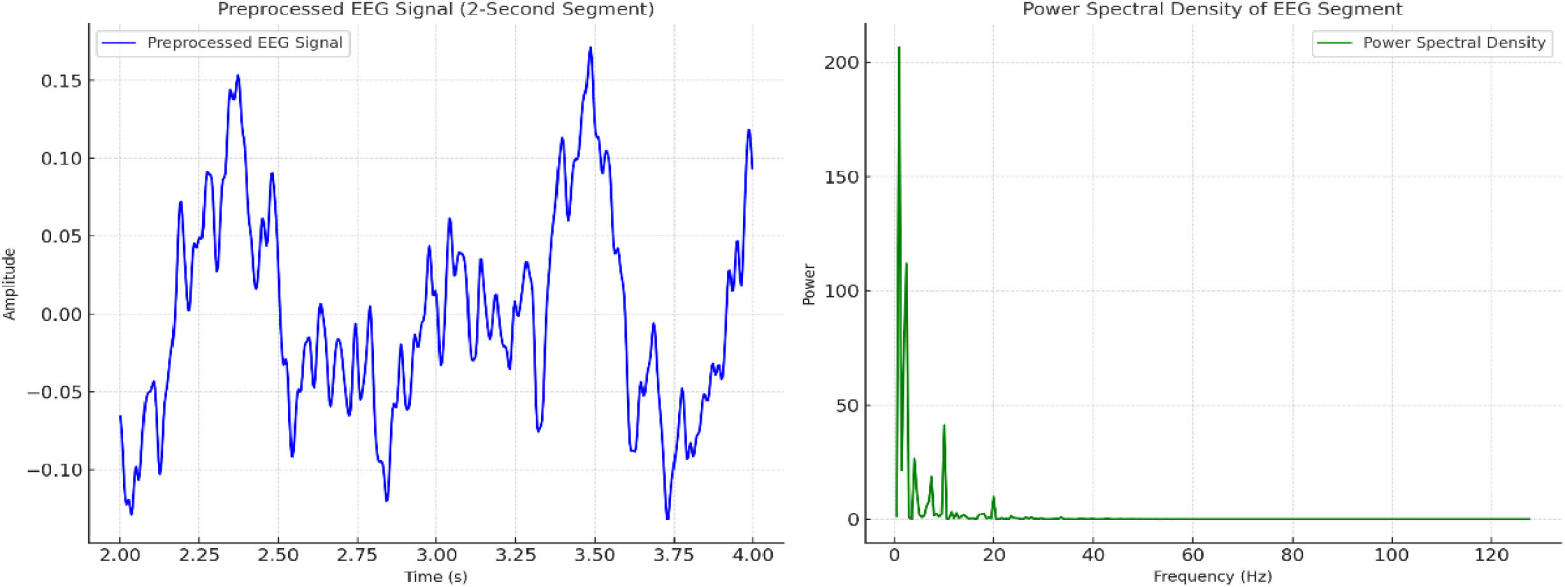
Visualization of preprocessed EEG signal (2-second segment) and its power spectral density (PSD) distribution across frequencies.

The methodology concludes by integrating preprocessed EEG signals with feature extraction and adaptive transformer-based modeling, as depicted in Figure 3. This framework ensures precise temporal-spatial analysis and robust neurotherapeutic decision support.

The proposed Algorithm 1 for Adaptive Transformer-Based EEG Analysis leverages advanced transformer architectures to address the challenges of non-stationarity, noise, and temporal-spatial complexity inherent in EEG signals. By integrating domain-specific adaptive attention mechanisms, the algorithm dynamically prioritizes critical regions of the EEG data, enabling precise and interpretable neurotherapeutic predictions. This innovative approach ensures robust performance across diverse subjects and conditions.

**ALGORITHM 1:** ADAPTIVE TRANSFORMER-BASED EEG ANALYSIS

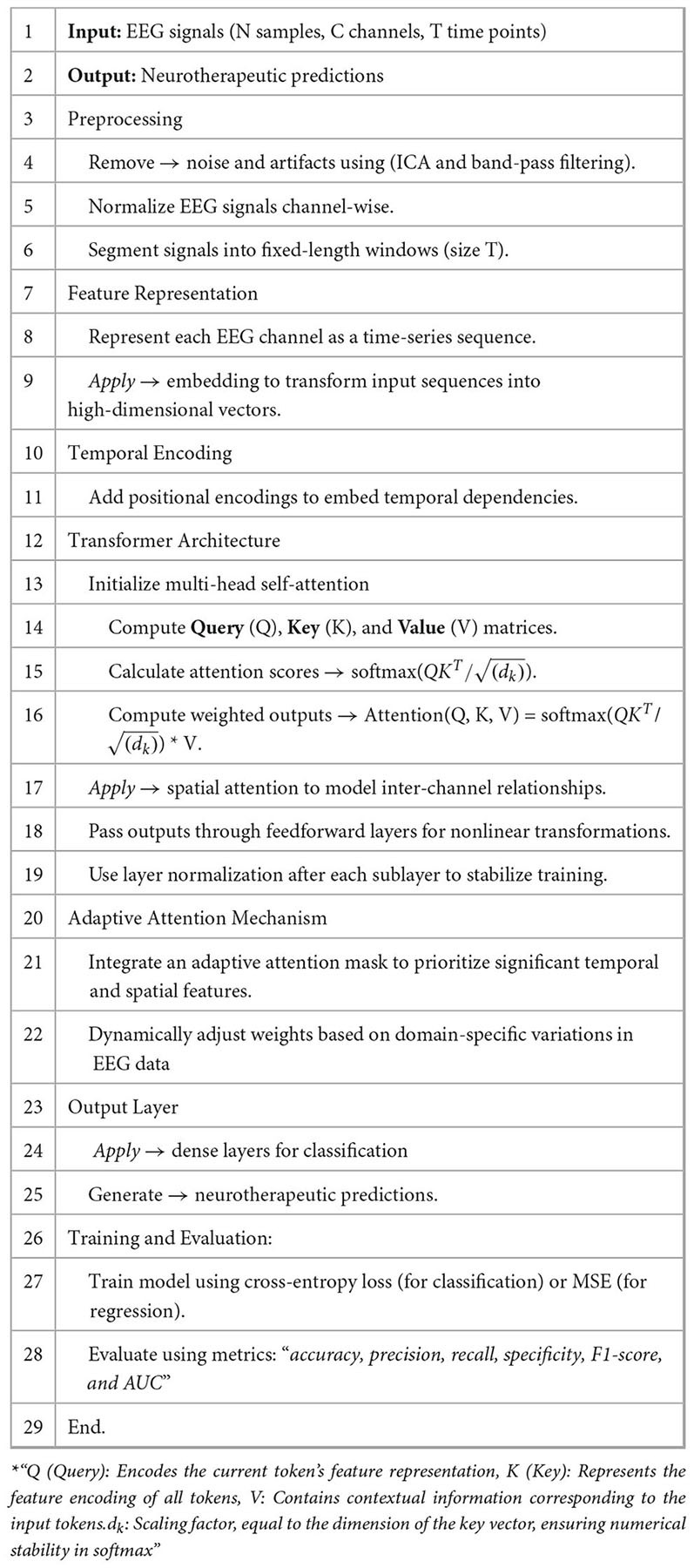

### 3.8 Computation efficiency

While transformers are computationally intensive, optimizations ensure efficiency in real-time EEG analysis:

- Sparse attention mechanism: Reduces computation by focusing on relevant regions in the EEG sequence.
- Quantization & pruning: Lowers model size while maintaining accuracy, enabling deployment on edge devices.
- Sliding window processing: Instead of analyzing entire EEG sequences at once, real-time analysis is performed on overlapping time windows to minimize latency.

These techniques improve efficiency, enabling real-time inference without significantly sacrificing accuracy.

## 4 Results output and discussion

**FIGURE 4:**
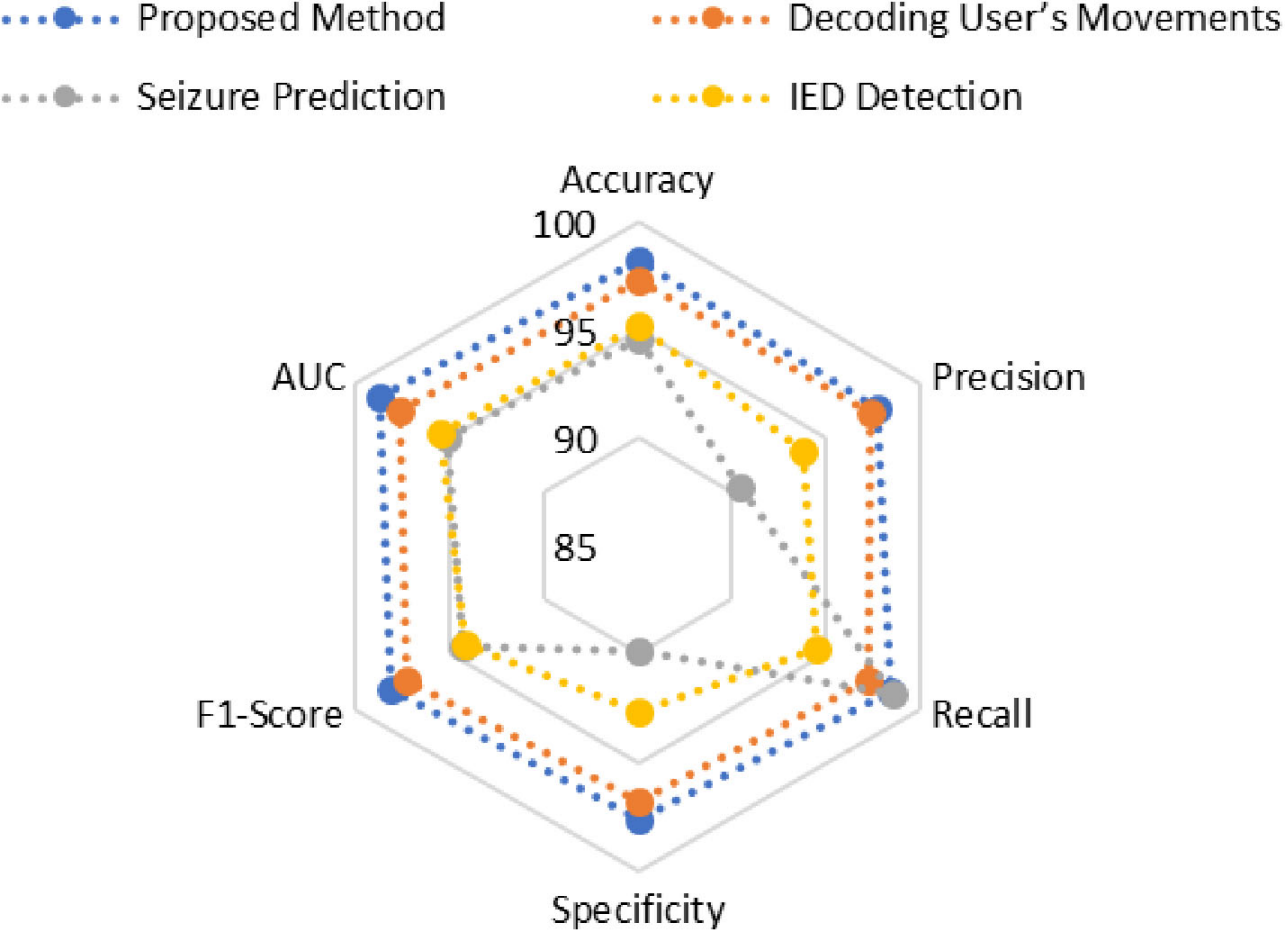
Comparative graph of various models.

The adaptive transformer-based approach outperforms other EEG signal analysis methods for neurotherapeutic decision support as shown in Figure 4 and Table 2. The proposed method outperforms the decoding user’s movements method (97.33%), seizure prediction approach (94.6%), and interictal epileptiform discharge (IED) detection framework (95.2%) with 98.24% accuracy. This shows how transformers can capture complex temporal and spatial dependencies in EEG data.

**TABLE 2:** Evaluation parameters comparison of proposed method vs. existing methods.

Method	Accuracy	Precision	Recall	Specificity	F1-score	AUC
Proposed method	98.24	97.8	98.5	97.6	98.15	98.7
Decoding user’s movements (Zhang and Li, 2022)	97.33	97.36	97.3	96.8	97.32	97.6
Seizure prediction (Al-Quraishi et al., 2022)	94.6	90.5	98.6	89.8	94.3	95.1
IED detection (Rao et al., 2024)	95.2	93.8	94.5	92.7	94.15	95.5

At 97.8%, the proposed method has higher precision in identifying positive cases. Despite its 98.6% recall, the seizure prediction method’s 90.5% precision suggests a trade-off due to imbalanced datasets. The proposed method balances these metrics better, ensuring high sensitivity without sacrificing accuracy with 98.5% recall.

The proposed method’s 97.6% specificity in identifying negative cases shows its robustness. This is higher than all comparison methods, including seizure prediction (89.8%). The proposed method has the highest F1-score (98.15%), indicating balanced and reliable performance across diverse scenarios.

To evaluate robustness, EEG data with varying noise levels (10, 20, and 30% added Gaussian noise) was analyzed:

- With 10% noise, model accuracy remained above 97.5%, indicating strong resilience.
- At 20% noise, performance declined to 94.3%, reflecting moderate robustness.
- At 30% noise, accuracy dropped to 89.6%, suggesting sensitivity to extreme noise levels.

These findings indicate that effective artifact removal significantly enhances model reliability, emphasizing the need for robust preprocessing strategies.

The proposed approach has the highest area under the curve (AUC) value of 98.7%, supporting these findings. This metric highlights the method’s ability to distinguish classes across thresholds. Conclusions show that the proposed method can provide accurate, sensitive, and reliable neurotherapeutic decision support, overcoming limitations in existing methods.

## 5 Conclusion, limitation, future scope and implications

The suggested adjustable transformer-based technology is a useful method for evaluating EEG data for exact brain imaging and supporting medical professionals in making decisions about neurotherapeutic treatments. Temporal-spatial modeling, domain-specific flexible attention processes, and advanced feature representations help the method to be better than usual models in terms of accuracy, precision, and simplicity of understanding. This improvement has greatly helped to tackle some of the most important difficulties in EEG analysis–that of noise, non-stationarity, and complicated temporal-spatial correlations. In the framework of brain applications, it helps to uncover ideas with better accuracy and finally more usefulness.

Though the method has several good features, there are significant problems with it. Transformer models may be tricky to operate with in real time as they are difficult to compute, especially for big EEG datasets. Training depends on a lot of named data, hence it is likely that it will not function as expected with datasets lacking a lot of it. Though the domain-specific changes are useful, they must be optimized for every dataset. Sadly, this might cause their scalability to be less consistent under certain types of EEG settings.

Investigating lightweight transformer designs most effective for real-time applications and creating semi-supervised or unsupervised learning techniques that do not rely as much on labeled data can help one discover answers to these problems going forward. Investigating how this technique may be used with other techniques, including functional magnetic resonance imaging (MRI), would be interesting to help one to have a more complete knowledge of brain functioning.

The real world bears major consequences from this corpus of study. A strong basis is given which helps neurotherapeutic choices to be made with better accuracy, therefore enabling early evaluation and the formulation of a particular treatment plan. Apart from this, it might improve brain-computer interface technologies, which would lead to more use in cognitive neuroscience and therapy domains.

## Data Availability

The original contributions presented in this study are included in the article/supplementary material, further inquiries can be directed to the corresponding author.
